# Beyond the hype: ‘acceptable futures’ for AI and robotic technologies in healthcare

**DOI:** 10.1007/s00146-023-01659-4

**Published:** 2023-04-22

**Authors:** Giulia De Togni, S. Erikainen, S. Chan, S. Cunningham-Burley

**Affiliations:** 1 The University of Edinburgh; College of Medicine and Veterinary Medicine; Molecular, Genetic and Population Health Sciences; Usher Institute; Centre for Biomedicine, Self and Society, 23 Buccleuch Place, Edinburgh, EH8 9LN United Kingdom; 2 University of Aberdeen, School of Social Science; Department of Sociology, Aberdeen, AB24 3FX United Kingdom

**Keywords:** Hype, AI, Robotics, Healthcare, Technoscientific imaginaries, Expectations

## Abstract

AI and robotic technologies attract much hype, including utopian and dystopian future visions of technologically driven provision in the health and care sectors. Based on 30 interviews with scientists, clinicians and other stakeholders in the UK, Europe, USA, Australia, and New Zealand, this paper interrogates how those engaged in developing and using AI and robotic applications in health and care characterize their future promise, potential and challenges. We explore the ways in which these professionals articulate and navigate a range of high and low expectations, and promissory and cautionary future visions, around AI and robotic technologies. We argue that, through these articulations and navigations, they construct their own perceptions of socially and ethically ‘acceptable futures’ framed by an ‘ethics of expectations.’ This imbues the envisioned futures with a normative character, articulated in relation to the present context. We build on existing work in the sociology of expectations, aiming to contribute towards better understanding of how technoscientific expectations are navigated and managed by professionals. This is particularly timely since the COVID-19 pandemic gave further momentum to these technologies.

## Introduction

AI methods and robotics are being used to support clinicians, surgeons and healthcare providers with decision making, to reduce error and improve diagnosis, treatment choice, and patient outcomes (Di Ieva 2019). These technologies are aimed at increased efficiency while tailoring healthcare to individuals’ specific characteristics and needs, and making healthcare more affordable and accessible (Mohd 2019). Health data are increasingly captured through electronic medical records (EMR) and new smart devices for individual use (Schüll 2016), and analyzed by AI deep learning systems to forge novel research agendas and provide personalized care. While expectations continue to rise about the future of medicine and healthcare promised by data-intensive innovation (Erikainen and Chan 2019), there is an urgent need to define and address the opportunities, challenges, and practical implications of using AI and robotics in healthcare (Hollis et al. 2019). This need has been further heightened by the COVID-19 pandemic context, which intensified the promotion of AI and robotic technologies to meet the demands of the health crisis (Jecker 2020).

In both public and scientific discourses, AI and robotic technologies have attracted high expectations around their imagined near-future capabilities, including both utopian and dystopian visions of technologies sophisticated enough to replace even highly skilled human workers in health and social care (Vicsek 2020). This is part of a wider discourse around the future of work and what a proliferation of AI and robotic technologies would mean for employment. Vicek (2020) has shown how these debates tend to cluster around polarized negative and positive predictions: on the one hand, these technologies are predicted to take over the human workforce, resulting in a dystopian future of large-scale unemployment; on the other, they are expected to enhance and assist rather than replace the human workforce, and although some jobs may become redundant, new jobs will be created. In both cases, the envisioned futures involve highly sophisticated automation of work that has not yet been developed (ibid). In the health and care context, despite the ‘AI hype,’ evidence remains scarce for successful implementations of AI products and robotics in clinical care.

The development of AI and robotic technologies also takes place in the context of neoliberal modes of governance adopted across different national contexts, including privatization and cuts to public funding combined with political rhetorics that promote citizens’ responsibility and control over their own lives (Brown and Barker 2012; Lorenzini 2018). While often framed as empowering for citizens, the consequence can be transfers of responsibility over health and wellbeing from states and public healthcare systems to citizens and patients, whereby the latter are encouraged to foster self-sufficiency (Brown and Barker 2012). AI and robotic technologies promise to enhance and assist individuals’ self-sufficiency, for example through technologies facilitating independent living and mobility for older adults and people with disabilities, in ways that can feed into and lend support for the neoliberal discourses.

In this wider context, AI and robotic technologies are transforming the relationships between people and machines in new affective, embodied and relational ways (De Togni et al. 2021). The health and social care contexts are particularly pertinent to examine such developments. This article explores interview accounts of AI and robotic innovation in health and social care from a group of 30 scientists, clinicians and other stakeholders based in the UK, Europe, USA, Australia, and New Zealand. Drawing on and extending existing theoretical frames within the sociology of expectations, we analyze the role of technoscientific expectations and visions of the future in shaping scientific and technological change around AI in the health and care sectors. We show how these stakeholders navigate a complex balance between high and low expectations, and promissory and cautionary visions of the present and future of AI and robotic innovation. They do so in ways that are simultaneously cautious and sceptical towards the high expectations associated with ‘AI hype’ while recognising the role of the promissory in appealing to the hype, at least strategically, as instrumental in gaining (symbolic and material) investment in AI and robotic research.

Despite their cautionary approach towards the AI hype, our participants are also direct contributors to the hype. This contradiction frames their complex navigations between high and low expectations, forming the background and basis for their construction of their own visions of ethically ‘acceptable futures’ for AI and robotics. These visions reflect the political and economic context in which AI and robotic innovation takes place, and they are framed by normative considerations about the kinds of futures that our participants would be willing to accept and about the ethical as well as technological limitations of the technology in the present context. We call this normative character an ‘ethics of expectations.’ We aim to contribute towards a better understanding of the dynamics of technoscientific expectations, by interrogating the metaphors and practices through which the future of AI technologies is mobilized as an object of present-day action and agency (Brown et al. 2000) by those who create and employ these technologies.

## Methodology

This research is part of an exploratory study examining different aspects of AI and robotics applications in health and care, focussing in particular on robotic surgery, digital pathology, and socially assistive robots (Authors 2021). Our analysis draws on 30 semi-structured, in-depth, one-to-one qualitative interviews with three groups of professionals: scientists, clinicians, and stakeholders using these technologies. In particular, participants comprised: (i) 18 scientists investigating and developing AI and robotics applications for health and care, including experts in computer science, health data science, and digital mental health prevention research; robotic engineers developing social robots; neuroscientists, psychologists, and academic clinicians; (ii) 4 clinicians including surgeons currently using robots such as the Da Vinci Intuitive System (an endoscopic system for minimally invasive surgery) and physicians using exoskeletons and robotic prosthetics for rehabilitation; and (iii) 8 stakeholders from the innovation industry including CEOs of IT companies and those working for the non-profit care sector, e.g. in elder care. To protect their privacy, all participants have been anonymized.

The interviews lasted 60 to 90 minutes, took place between February and August 2020, and were audio-recorded and transcribed. Five interviews were carried out in person in British cities and 25 remotely via online platforms. The interviewees were based at leading institutions in the UK, Europe, USA, Australia, and New Zealand. After preliminary discussion with colleagues in the field, we used snowballing to access a network of stakeholders. Our group of interviewees is heterogenous, from a variety of backgrounds, and not necessarily representative of the field as a whole. Nonetheless, it constitutes an important professional grouping, actively contributing to research and decision making in the field. Nearly two thirds of the interviewees can be considered elite scientists because of their prolific research careers including contributions to influential journals and international media. These elite scientists had slightly more pessimistic views on the technology than other participants.

During the interviews, our respondents were asked about the impact of these new technologies on health and care practices, especially in the fields of surgery, pathology and care, and on society more widely; their personal views and experiences of using these technologies as opposed to how these are portrayed by the media and public understandings; and their hopes and visions for the future of these technologies. The interview transcripts were analyzed using inductive thematic analysis (Braun and Clarke 2008) to identify patterns of meaning in the semantic context. The data were initially coded to identify key features, which were then iteratively grouped into themes of wider patterns of similarity. These main crosscutting themes are grouped into the two sections that follow: (i) balancing high versus low expectations, and promissory versus cautionary discourses on the current state of these technologies; and (ii) navigating the human–machine relationship in the construction of ethically ‘acceptable futures.’

We follow Coeckelbergh’s (2020: 28) suggestion to go ‘beyond the hype’ and not limit the analysis about AI to “dreams and nightmares about the distant future.” Consequently, we critically examine the interview accounts in relation to assumptions about AI and the human, while looking more in detail at what is said about existing AI and robotic applications. There are limitations to the study as we did not talk to caregivers or care recipients, focussing instead on our relatively small group of stakeholders. Our research was limited to English speakers from high-income economies. Future studies should involve users and cross-cultural comparison.

### Constructing expectations

Our analysis builds on sociology of expectations research that documents the role of future-oriented discourses and promissory narratives in the politics of science and innovation (Brown and Michael 2010; Tutton 2011; Gardner et al. 2015). These expectations have been identified around AI technologies in different areas of social life (Vicsek 2020) and have a temporal character whereby visions for the future are attached to technologies as their ‘promise’ (Brown et al. 2000). Such expectations are performative: they shape and guide science and innovation, attracting interest and investment (Borup et al. 2006). Expectations are, therefore, a constitutive force in science and innovation, harnessing economic, cultural and symbolic capital and enrolling different actors, including funders, in support of particular research and innovation agendas (Broer and Pickersgill, 2015). Technoscientific expectations tend to go through cycles of hype, which attract new actors/investments into an area of research, yet pose challenges for innovators (Bakker and Budde 2012). This is because hyped expectations tend to become overly optimistic, causing standstills once the hype has subsided (ibid).

While much of the sociology of expectations has focused on optimistic promissory futures, some has considered the role of low expectations in the innovation process, including modest visions of more uncertain futures (Pickersgill 2011; Tutton 2011). Low expectations help manage the tensions between hyped future visions around innovation and the exigencies of implementation: “the dynamism of innovation emerges from a complex intertwining of low and high expectations; an interplay of promise, hope and optimism, and uncertainty, pessimism and ambivalence” (Gardner et al. 2015: 1003). Moreira and Palladino (2005) identify two conflicting but interlocking organisational logics within discourses around new and emerging scientific developments, namely the ‘regime of truth’ and the ‘regime of hope.’ The former entails “an investment in what is positively known, rather than what can be,” whereas the latter is characterised by “the view that new and better treatments are always about to come,” with research and development justified by the “promise of finding miraculous cures” (ibid: 67).

Fitzgerald (2014, 2017) and Pickersgill (2011) suggest that scientists and clinicians occupy an intermediate position between these two regimes and can recalibrate expectations to protect their professional reputation and career trajectories, while ensuring that innovation is not stymied by its failure to live up to the hype. Fitzgerald (2014: 241) describes the ambivalence experienced by scientists navigating the intermediate terrain of an evolving biomedical field characterized by unknowns as “entangled registers of both promising hope and deflated uncertainty.” The neuroscientists interviewed by Fitzgerald admit to the “fluffiness” (2017: 42–43) of their research, allowing intuitions and the “softly subjective” (ibid.: 135) not only to inform their diagnoses but also to enable them to continue in their pursuits despite multiple uncertainties. Relatedly, while the optimistic future-oriented rhetorics of hype continues to animate innovation projects through narratives of “breakthrough” and “discovery,” in practice stakeholders often use a kind of “epistemic modesty” (Will 2010) to focus on what is realistic.

Expectations have a normative character, shaping their dynamics, and how different actors navigate the relationship between ‘hope’ and ‘truth’ under conditions of uncertainty. Expectations around technological innovations often express desirable futures reflecting the hope and promise of future capabilities. Yet, concerns and caution about the risks the technologies carry and their possible failure in both practical and ethical terms run parallel to such promissory visions (Borup et al. 2006). Normatively framed tensions between hope and promise, and concern and caution, pertain both to the present and future of these technologies and reflect contestations over questions around what the technology should and should not to look like as well as what is and is not actually realisable (Erikainen and Chan 2019). This normative character of ‘should’ imbues expectations around AI and robotics technology with an ethical and value ‘baggage’ that shape these future visions.

We refer to these normative, temporal dynamics as ‘ethics of expectations,’ where the notion of ‘ethics’ is intended specifically to capture the centrality of the ethical and value baggage when it comes to how our participants conceptualised AI and robotics innovation. The ethics of expectations concerns the normative character of the envisioned futures and the challenge of balancing the potential harms and ethical shortcoming of these technologies in the present context with the hype and promissory expectations. The ways in which our participants discursively negotiated tensions between present harms and future promise illustrate the difficulties for stakeholders in taking a critical stance towards these technologies, while continuing to work on and with them in a context where the promotion of promissory expectations is important for attracting investment in their work. Ambiguity was a rhetorical strategy to legitimate stakeholders’ work and immunise it against critique. This also raises questions about the extent to which being ambiguous or realistic about the nature and potential of AI is a form of pre-emptive ethical self-regulation.

As will be seen from the accounts of our participants, the AI and robotics innovation process in healthcare poses a challenge of creating a culture of responsible innovation that invokes ‘ethically acceptable futures’ for these technologies. One of the main challenges facing the data driven society is how to keep the human in the loop while shaping AI and robotic systems within a positive culture of ethics and the symbiotic collaborations between humans and machines. This inevitably raises questions of responsibility, intentionality, legitimacy, and acceptability of these technologies. Thus, building on Brown et al. (2000), we contend that one should look not *into* the future but rather *at* the future and how it is mobilised as an object of expectations today to understand the nature and dynamics of AI and robotics innovation.

### Balancing high versus low expectations

As Hedgecoe (2003) argues, terminological choices around a field of scientific research have performative implications. These include attracting funding for activities within the field and gaining support for research and development. This process is complicated in the context of AI and robotics by the interdisciplinary and multifaceted nature of the research area, including contestation over what, exactly, should be included within its remit.

To understand this complexity, we started our interviews by asking participants to provide a definition of AI. Interviewee 7 (senior professor) who has worked in the field for more than 30 years, laughed at this question, commenting that “asking for a definition of AI is asking for the moon.” Similarly, Interviewee 11 (early-career researcher) lamented that they were “annoyed because AI has become a jargon for a whole range of technologies […] thus, it is difficult to understand what people are actually referring to when they use the word AI.” In the words of Interviewee 15 (mid-career professor) “AI is like a salad bowl of different technologies” including machine learning, computer vision and natural language processing. Instead of using the term AI, some preferred ‘augmented intelligence’ or ‘intelligence amplification,’ arguing that ‘artificial intelligence’ is ‘not meaningful’ because it only creates confusion. Others preferred to limit their definition to the differentiation between ‘strong AI’ and ‘weak AI,’ stressing only the latter exists today. While professionals working in the field disagree over what AI actually entails, much of the hype around AI research focuses on ‘strong AI’ and intelligent machines, which are sometimes imagined capable of surpassing human intelligence.

Associating the need to stabilise terminology, interviewees focussed on the practicalities of developments in the here and now, thus attenuating expectations. The high expectations of AI mirror previous hypes around advanced technologies such as industrial robots and machines, nuclear technology, nanotechnology, the internet and biotechnology (Cockelbergh 2020). As these technologies became embedded in everyday life, the hype deflated. Relatedly, interviewee 2 (senior professor and clinician) noted:

I personally don’t care if we call it AI or technology or machine learning. […] In my research group, most of us understand that these terms are sort of metaphors or maybe marketing terms of [laugh] some kind. […] After all the hype in this area, kind of, vanishes then we’ll be able to take those technologies that work and they will become incorporated into things and we won’t notice them. There will be no big, singing, dancing, kind of, AI systems for everyone to see.

Interviewee 2 highlighted the unstable and contested nature of this research area and explicitly framed the question of terminology and definitions as metaphorical or “marketing” choices. This ‘marketing’ taps into the hype surrounding this research field, seen to influence innovation including through harnessing investment, but the interviewee also expected that the hype will eventually subside, leaving room for more realistic assessment of these technologies’ capabilities.

Many interviewees stressed the importance of being realistic about the nature and potential of AI and robotics, especially to resist the popular expectation that AI and robots will replace human actors in health and care–an expectation that has become embedded in the AI hype as its negative corollary. This expectation is part of the wider future of work discourse where, as noted above, AI and robots are predicted either to take over or enhance the human workforce. Our interviews tended to the latter perspective. Interviewee 16 (mid-career academic) warned that “there’s not that much magic that goes on behind AI.” He preferred referring to “IA” or “Intelligence Amplification,” stressing the potential of these technologies to enhance not replace human capabilities through, for instance, robot-assisted high-precision surgery. Through refining definitions of AI in this way, interviewees created more realistic and acceptable, from their perspective, versions of their work.

Some also highlighted the risks hidden behind both the promissory and more dystopian future imaginaries. These were often articulated in ethical and social terms: high expectations around future AI innovation can function to side-line more pertinent ethical and social implications of these technologies now. Interviewee 20 (senior data scientist), referring to algorithms for diagnostics, lamented that the AI hype, including the speedy adoption of related technologies without due attention to their ethical and social implications, has resulted in a “tremendous amount of poorly designed systems” which pose problems for ethics and fairness due to e.g., gender and racial biases.

Interviewee 7 (senior scientist) highlighted the importance of investing in human carers rather than in technological applications: “Do not idealise the technology, do not believe the hype, pay human carers more. Don’t try to replace humans with robots.” Interviewee 7 defined themselves as ‘pragmatic,’ and admitted that they did not fully trust Socially Assistive Robots (SARs) despite having spent their entire career studying them. They supported the idea of a “care-assistant robot” for older people living alone, which could be helpful in e.g., bringing water, reminding when to take a medication, and detecting a fall; but highlighted that these technologies should be developed, “not to replace but to assist human carers.” Indeed, the phrase “not to replace but to assist/enhance/augment” was a recurrent one.

While our interviewees generally made considerable efforts to caution against the AI hype, the hype itself was also explicitly framed by some as carrying the benefit of increasing investment and funding in AI and robotics development and, thus, advancing their own careers. This was articulated especially in relation to the need to attract and secure research funding. Amongst scientists, early career researchers in the process of applying for major grants felt pressure to convince funding institutions that their research was meaningful, leading to societal impact. The interview accounts revealed how they felt compelled to promote, create and sustain systems that would demonstrate the promise of these applications. This group was particularly fearful of a drop in AI and robotics research funding if these technologies fail to meet current high expectations, resulting in an ‘AI winter,’ or what Interviewee 1 (mid-career scientist) termed “the valley of despair.” Indeed, as we noted above, technoscientific expectations tend to incite ‘hype cycles’ where the peak of positive or inflated expectations is followed by disillusionment and possible standstills in innovation once the hype subsides (Bakker and Budde 2012; Woodie 2015).

In contrast, more senior scientists who had already secured research funding or tenure positions were generally more openly sceptical of the hype around AI, and more likely to highlight the currently limited nature of the technologies they were researching. For example, Interviewee 15 (mid-career scientist), who was studying Socially Assistive Robots (SARs), stated:

I don’t see the immediate impact right now in my work, but the things like predictive stuff [algorithms for diagnostics] that’s beginning to happen in modelling and comparison, that has real potential. I think we’re several years, you know, five or six years away from […] people understanding [SARs’] potential, really.

The same interviewee also described the hype as a “marketing gimmick, a lot of the time,” stressing how nowadays digital pathology has the highest potential to generate benefit in medicine, but the hype tends to focus more on SARs as ‘intelligent machines.’ They admitted that such hype was the reason they obtained substantial funding for their research. Most interviewees (especially senior scientists and clinicians) saw the highest potential in applications such as digital pathology, diagnostics, telemedicine, and wearable devices collecting health data used for personalized/preventive medicine. However, they also considered that within the ‘AI hype,’ physically and socially assistive robots are considered ‘cooler’ and more appealing to businesses and governments. Interviewee 19 (senior scientist), for instance, commented on how the use of exoskeletons and robotic prosthetics had transformed their field:

The field of rehabilitation it’s now become cutting edge because of this [the use of exoskeletons and robotic prosthetics]. It was kind of a dirty field, like nobody really cared about this field because it’s dealing with people with disabilities and ‘why don’t they get a walker or a cane or some wheelchair?’ and nobody cares. It’s kind of a boring field. Even in hospitals literally we were in the basement of the universities and hospitals, because it’s kind of a stigmatic field. It’s all these disabled people... They just didn’t want them to be the big front part. Now we’re the front-facing.

Interviewee 30 (surgeon), who was using the Da Vinci Intuitive System, also noted that their patients appeared more confident about undergoing surgery if a robot was involved:

I mean, one reaction was [laughs] from a son of a patient when we were discussing. He said, well, you should have that [robotic surgery], mum, because that means it’ll be better than the standard. So, he sort of didn’t quite twig that it was nevertheless still entirely under my control. I think it does augment my ability to do the surgery more easily so it makes it easier for me; but I’m not sure it necessarily reduces the chance of specific complications because, well, often those are not necessarily intraoperative; they are to do with recovery and more to do with patients’ existing comorbidities than what happens technically.

The scientists and clinicians we interviewed navigated a complex balance between high and low expectations. They recognised that AI and robotics hype had in many ways facilitated their research through functioning as a marketing tool and pushing investments in the sector, and they themselves constructed promissory expectations about the future of related technologies. Concurrently, however, respondents were concerned about unrealistic expectations and their potentially damaging implications, including in relation to funding and innovation standstills that could result from the hype’s deflation. These concerns were also framed in terms of normative considerations that incited reflections on the ethics of expectations, including the extent to which hyped expectations distract from due attention being paid to the ethical and social implications of these technologies including investment in human carers in the present context. In many ways, our interviewees navigated the balance between high and low expectations by taking an intermediate position between the ‘regime of truth’ and the ‘regime of hope’ (Moreira and Palladino 2005). They concurrently articulated Fitzgerald’s (2014: 241) ‘entangled registers of both promising hope and deflated uncertainty’ around AI and robotics, in ways connected with the strategic need to secure funding, the reputation of the research area, and their own career trajectories.

### The human–machine relationship: building ‘acceptable futures’

Our interviewees rhetorically used the present state of AI and robotic technologies to argue that innovation in the future may transform clinical practice in terms of order of magnitude and amount of data, but many framed the continuation of care provided by humans as important. This links with the issue discussed above and articulated by several participants, that AI and robots should not be expected to replace human caregivers but, rather, to provide an assistive or augmenting role. Interviewee 6 (senior academic) stated:

Some experts are predicting that we won’t need to interact with other people at all. I think that’s very unlikely to happen for the foreseeable future, especially in primary care. You probably know there is robust evidence that if someone does have a primary care provider, they do much better than if they don’t. The longitudinal nature of the relationship and the interpersonal part of the relationship seem to be pretty important.

The discussion surrounding SARs for elder care generated contested articulations of the future. Interviewee 9 (senior academic) lamented that “the problem with social robots is that people imbue them with more intelligence that they actually have.” Interviewee 20 (senior academic) warned: “I think there’s some confusion between the Hollywood ideas of robots and what robots can actually do. Robots are just machines.” Interviewee 12 (early career researcher) stressed the importance of “ensuring people are aware that a robot is not a human […] and let [ting] people have the choice to have a relationship with a robot.” Interviewee 2 (senior academic and clinician) dismissed the idea of care robots in these terms: “I think this is, erm, unlikely to happen and if it does happen it would be somewhat dystopian.” These narratives rendered a future where human care is replaced by machines as unlikely, undesirable, and unethical. In their articulations of ‘ethically acceptable futures,’ ‘better’ care can only be provided by human actors, as it is reliant on interpersonal human connections and relationships.

Interviewees with expertise in robotics described SARs as well-trained machines capable only of limited actions, far away from the idea of “robot doctors” (Chin-Yee 2019). Robots were framed not as ‘super machines,’ but as physical embodied agents having the capacity for mobility and to detect and avoid obstacles but not entailing interpersonal relationships of the kind provided by human caregivers. This was also the case for robotic surgeons, which are still highly dependent on the skills of human practitioners. SARs and robotic surgeons were often contrasted with diagnostic algorithms, which do not generally aim to replace humans but facilitate efficiency and faster workflows. These were generally recognised as technologies that are already ‘highly beneficial’ to clinical practice and ‘very promising,’ despite limitations especially in relation to algorithmic biases that are prejudicial to certain groups (see e.g., Eubanks 2018; Noble 2018).

Our interviewees articulated near-certain expectations of what the future should *not* be like, clustered around ethical and social concerns pertaining to the current state of these technologies. Interviewee 9 (senior academic) problematized it in these words: “I don’t think there’s enough thought so far been given to ethics, we’re terrible sometimes. We’re getting better as technologists but we’re very much of the ‘build it and then worry about stuff’.” In the case of digital pathology, a few participants commented that biases remain difficult to tackle because experts typically have little understanding of why AI systems make decisions or exhibit certain behaviors resulting from the so-called ‘black box’ problem of deep-learning models (Mittelstadt et al. 2016; Hall and Gill 2019).

Although expressing such concerns, many respondents still argued that society will eventually need to ‘adapt’ and ‘accept’ these technologies. These arguments were generally not geared towards simply embracing the idea of technologically-driven care as replacing human-provided care, but rather towards accepting AI and robotic technologies as an important assistive part of care or, as one participant remarked, ‘better than nothing.’ This was especially under conditions where human care provision is limited or direct human contact and connection may not be possible, such as health and care in the context of the COVID-19 pandemic. Interviewee 13 (stakeholder from the non-profit sector) questioned:

Would people be willing to be cared for by a robot in the last days of their life? […] We cannot replace human beings, especially in such circumstances. We need human contact. And, nevertheless, the COVID-19 pandemic is forcing many to do this: people are dying alone, without the opportunity to meet their family members and loved ones.

Despite emphasizing the importance of human connection, Interviewee 13 concluded that, in certain situations, care robots may become the only available option to provide care to vulnerable populations.

In June 2020, Aymerich-Franch (2020), editor of *Nature Machine Intelligence*, commented: “With physical distancing and isolation measures deemed critical to slow the spread of COVID-19, social robots have finally found an opportunity to demonstrate their real value in society.” Indeed, since the beginning of the pandemic the use of SARs in hospitals and care homes has increased in some countries to ensure socially distanced assistance (Jecker 2020). Some of our interviewees considered the COVID-19 driven enthusiasm toward SARs as a positive turn because they saw the societal value of their research finally recognized. Interviewee 29 (senior scientist) commented:

You don’t know how many people have told me since the pandemic started, ‘oh gee, you know, if you only had that friendly robot in your house sitting on your desk now, it would remind you also to, you know, wash your hands more and to wear a mask and also to connect with others so you’re not so lonely.’ And I was like, ‘yeah, exactly, if everybody had a friendly robot,’ which is what I’ve been saying for fifteen years, right?

Interviewee 10 (stakeholder from the industry) suggested that the COVID-19 pandemic has already transformed human interaction. They expressed hopes that researchers and healthcare practitioners would learn from these exceptional circumstances: “Lack of human connection can be tricky […] but the challenges of living on your own for a long time can be solved by these applications [SARs].” Interviewee 28 (surgeon/senior academic) argued:

I suppose we’re going to have chatbots doing diagnostics in the future. And I mean, it sounds awful… It sounds like it’s going to be really frustrating because they won’t understand what you say and they’ll get it wrong and, you know, you’ll complain of a sore head and they’ll tell you that you’ve got arthritis in your knee or something like that. But as these things become more sophisticated maybe there will be some screening, some triage of patients in order to help the health service. […] I’m very taken with the technology and I want the technology to be good and I want… You know, I think it is…I do think it’s the future.

Mirroring this cautious optimism and considering how society may eventually have to rethink human care and accept technologies like SARs, Interviewee 21 (senior academic) used the example of Japan, which has the world’s most rapidly ageing population and is actively using SARs as a solution to tackle this problem:

In Japan one of the uses they have of the robots is with elderly people who are often very isolated socially. And, you know, […] this is not a new thing. I mean, elderly people who live alone often leave the television on constantly, just to hear conversations, just to have a voice in the house. And so […] there’s nothing new about the idea of providing virtual human connection. It’s been done with radio and television for a very long time. There is part of me that wishes that [laughs] we didn’t need it and that we could have the kinds of communities where people feel socially connected, including people who are elderly and people who are socially isolated for other reasons. But, you know, if given that we can’t have a perfect society in that way, I certainly think it’s better if having a robot that you can talk to makes you feel less alone. I think that’s better than feeling lonely.

Many interviewees also considered AI and robotics are being promoted as a technological solution to solve complex socio-economic problems in support of specific financial and political interests. Particularly in the case of rapidly ageing societies, SARs are used to promote independent living amongst older people, ostensibly to free up overwhelmed, underpaid caregivers from performing exhausting tasks, instead of recruiting more human caregivers and paying them more. These ideas align with neoliberal governmentality and related discourses promoting the transfer of responsibility of caring for the self from the state to individuals’ practices of self-monitoring, self-governance, and independent living (see e.g., Lorenzini 2018). While our participants tended to emphasize the importance of human provided care and connections, the cautious optimism they articulated in relation to AI and robotics often focused on the potential of these technologies to compensate for gaps in health and care services, and to contribute towards individuals’ ability to care for themselves under constrained public support systems.

The above articulations of present and future challenges around the role of AI and the human–machine relationship in health and social care can be understood as visions of ‘acceptable futures.’ In articulating these futures, our interviewees expressed what would be ethically and socially acceptable and realistic. The key elements of this were the importance of ‘not replacing humans with robots’ and acknowledging that ‘we may have no other choice left,’ or ‘we can’t have a perfect society’ and technologically driven care may be ‘better than none.’

One interviewee went further and expressed their willingness even to be “replaced by a robot” if this may benefit society, albeit with caveats. Interviewee 5 (surgeon/senior academic), discussing robotic surgery, noted that: “If you lose that human connection [in healthcare provision] then I think it becomes less rewarding but also, I think more difficult for patients to know who to trust.” They then added:

I would be delighted to be replaced by a robot if it turns out that artificial intelligence can do my job better than I can. […] There sometimes is this tension, I think, between AI and the workforce and whether, you know, it’s going to have a detrimental effect on your ability to sustain your job and your livelihood in the future and I don’t see that. I’d be delighted to be replaced by a robot.

Organizational narratives are increasingly framed by the need to secure the ‘latest’ AI and robotic systems to provide better service, drafting strategic road-maps which anticipate future-oriented paths for the sector. However, Interviewee 5’s embrace of the potential of AI and robotic technologies was still conditioned by the realization of the technology’s unrealized promise to do the job better than a human surgeon can, as well as an acknowledgement of the importance of human connection and trust. As this example shows, in constructing their own visions of the future of AI and robotic technologies, our participants were making sense of the human–machine connection in the context of the increasing infusion of AI and robotic technologies into health and care. Nonetheless they maintained the importance of interpersonal relationships and human connection in care provision. The ‘acceptable futures’ that they constructed were plural, contested and often characterized by uncertainty. Such visions generally reflected a perceived need for a balance, not only between being realistic and being hopeful about these technologies, but also between endorsing them and endorsing the value of human care and relationships.

As those interviewed for this study worked in the AI field and thus in many ways directly contributed to the hype around AI and robotics, including by promoting the future promise of this research area to gain funding, the ethical viewpoints they expressed in the interviews were more nuanced and cautious. In navigating high and low expectations and hype and caution around these technologies’ potential, they constructed an ‘ethics of expectations’ in relation to what these technologies can do in the present and in the futures that they envisioned. High expectations and promissory narratives around AI and robotics were conditioned by the present ethical as well as technological limitations of these technologies. Concurrently, the role(s) that were attributed to AI and robotics in the future were conditioned by the normative questions of what would (or should) be ethically and socially desirable as well as what is realizable. This ethics of expectations balances precaution against pro-action, tempering techno-positive enthusiasm for the potential of AI and robotics with concerns for ethical values and what might be side lined or lost. Respondents were seeking to mitigate foreseen ethical and social harms of these technologies and to avoid the pitfalls of the “hopeful principle” and “automatic escalator” (Holm and Takala 2007) in over-promising on their potential. The interviewees in our study seemed alive to this dilemma, keeping the human, affective and embodied dimensions in their framings.

## Conclusion

In this paper, our aim has been to build on the existing body of research in the sociology of expectations to examine the strategies that scientists, clinicians and other stakeholders in AI research and innovation use to navigate and construct visions about the future of AI and robotic technologies in healthcare. We focused on those engaged in surgery, pathology and social care to represent contrasting developments and applications. We documented how high expectations and promissory visions associated with the future of AI and robotic applications in health and care have created challenges for scientists and clinicians, who work to balance these promissory visions with cautionary accounts of more realistic expectations. The hype was perceived as advantageous in providing momentum to research innovation projects by attracting symbolic investment and financial resources. This speaks to the performative power of high expectations around technoscientific innovation, as there is strategic value in endorsing and celebrating high expectations to attract investment. It should also, however, be contextualized in relation to the wider context of neoliberal modes of governmentality that that promote increasing citizens’ responsibility over their own lives, including via technological means.

Our participants also engaged in balancing acts between high and low expectations, and drew boundaries in their efforts to distance themselves from the hype while maintaining credibility for their research and practice. Over-optimistic visions of the future were perceived as dangerous in creating false hope about the power of AI and robotics and unrealistic expectations of performance, which may harm AI and robotics research and innovation through deflated investment if the outcomes of this innovation fail to match expectations. We showed how our participants negotiated the tension between sustaining and nurturing the hype while calling for the recalibration of expectations, integrating these technologies into an ethically and socially responsible framework.

We have shown how different stakeholders constructed their own visions of ‘acceptable futures’ that were centrally concerned with the changing nature of human–machine relationships. The construction of these futures involved negotiation and balancing between different demands, including the notion that AI and robotic technologies should not be seen as replacements for human care, but also that technologically driven care is at least ‘better than nothing.’ This was seen to pertain especially to contexts of limited health resources and increased demand for health and care services, such as during the COVID-19 pandemic where AI and robotic innovation was promoted as an important part of service provision to meet such needs. Through this balancing of different social and ethical as well as technoscientific demands, the participants articulated their perceptions of futures that would, or should, be ethically and socially acceptable, as well as realistically achievable, if not the most ideally desirable. Their articulations of both the present and future potential and limitations of AI and robotics technologies were thus framed by an ethics of expectations that positioned normative considerations as central to how the expectations were expressed.

In conclusion, we hope to have contributed towards an understanding of the complex dynamics underpinning technoscientific expectations by showing how scientists, clinicians and other stakeholders from the industry and non-profit care sectors articulated and navigated a range of expectations towards AI and robotic technologies in the areas of health and social care. They did so in nuanced ways that generally resulted in an intermediary position between high and low expectations and promissory and cautionary discourses. This intermediary position, in turn, foregrounded how respondents constructed their own visions of such socially and ethically acceptable futures as part of their efforts to craft a place for themselves and the AI research and innovation field. In doing so, they worked to consider not only the potential of AI and robotics, but also wider social and ethical issues around human–machine and human–human relationships, and the challenges facing the health and care sectors as a whole. The trajectory of future AI and robotic innovation in health and social care will continue to reflect and be shaped by these discourses, hence the importance of further explorations in this area.

## Data Availability

The datasets generated during and/or analysed during this study are not publicly available to protect study participant privacy.
